# Effect of Dexrazoxane and Amifostine on the Vertebral Bone Quality of Doxorubicin Treated Male Rats

**DOI:** 10.2174/1874325000802010115

**Published:** 2008-07-14

**Authors:** F Mwale, G Marguier, J.A Ouellet, A Petit, L.M Epure, J Antoniou, L.E Chalifour

**Affiliations:** 1 Lady Davis Institute for Medical Research, SMBD - Jewish General Hospital; 2 Division of Orthopaedic Surgery, McGill University; 3 McGill Spine & Scoliosis Unit, Montreal Children Hospital; 4 Division of Experimental Medicine, McGill University, Lady Meredith House, Montréal, Canada

## Abstract

Doxorubicin (DOX) is widely used in combination cocktails for treatment of childhood hematological cancers and solid tumors. A major factor limiting DOX usage is DOX-induced cardiotoxicity. However, it is not known whether protectants like dexrazoxane (DXR) and amifostine (AMF) can prevent DOX-mediated bone damage. The present study investigated whether administration of AMF alone or in combination with DXR would prevent any DOX-mediated bone damage. Male rat pups were treated with DOX, DXR, AMF, and their combinations. On neonate day 38, the bone mineral density (BMD), bone mineral content (BMC) and the micro-architecture of the lumbar vertebrae were analyzed. We have shown that when male rats are treated with DOX, DXR, DOX+DXR, AMF, DOX+AMF or DOX+DXR+AMF, there is a decrease in lumbar vertebral BMD (p<0.05). Furthermore, the relative bone volume (BV/TV) was decreased by DXR, DOX+DXR, and DOX+AMF treatments. Interestingly, DOX+AMF significantly increased BV/TV when compared to DXR treatment (p<0.04). The trabecular number (Tb.N) decreased with DXR and DOX+DXR and increased with DOX+AMF treatments. This information will be useful in designing better cancer combination therapies that do not lead to vertebrae deterioration.

## INTRODUCTION

The strength of adult bone reflects factors that regulate bone quality (architecture) and density (bone mass or quantity of calcium deposited/unit of bone) acquired during childhood and adolescence. Near-maximal or peak bone mass of the vertebrae and femurs is achieved at the completion of pubertal development [1]. Bone mineral density (BMD) and bone mineral content (BMC) measurements using dual-energy X-Ray absorptiometry (DXA) are widely used to measure bone density and determine fracture risk for osteoporosis [2-5]. Quantitative assessment of micro-architectural characteristics, such as relative bone volume (BV/TV), trabecular number (Tb.N), trabecular separation (Tb.Sp), and micro computed tomography scanner (Micro CT) are used to estimate bone strength [3].

Chemotherapies administered to children with cancer have the potential for long-term negative effects on bone [6] and can cause osteopenia during treatment [7]. The reduced height and mineralization, and the increased bone fragility found in cancer survivors can be caused by several factors that include nutritional deficiencies, the malignancy itself, radiation, corticosteroids, and direct action of anti-cancer drugs on bone [7, 8]. Current treatments of childhood hematological cancers and solid tumors can involve the anthracycline antibiotic doxorubicin (DOX, Adriamycin™) in combination cocktails [9, 10]. DOX-induced cell killing is due to increases in oxidative stress causing apoptosis, as well as to its ability to bind and stabilize DNA topoisomerase II cleavable complexes [11, 12]. A major factor limiting DOX usage is DOX-induced cardiotoxicity. Indeed, some studies suggest that young females have a greater incidence for DOX-induced cardiotoxicity than young males [13-15]. Dexrazoxane (DXR, ICRF-187, Zinecard™), also known as (+)-1,2-bis (3,5-dioxopiperazinyl-1-yl) propane, is the water soluble S-(+)-enantiomer of the racemic razoxane (ICRF-159) and was originally designed as an anti-cancer agent [16, 17]. DXR can reduce or prevent DOX-induced cardiotoxicity in children [18-20]. Amifostine (AMF, Ethyol™) is a cytoprotective agent that protects a broad range of normal tissues from the toxic effects of chemotherapy and radiotherapy without attenuating the tumor response [21].

The effect of DOX, with or without DXR and/or AMF, on bone in children is not well understood. We have previously shown a significant decrease in the right femoral and lumbar vertebral BMD of female rats treated with AMF, AMF+DOX or AMF+DXR+DOX [22]. However, the effect of AMF alone or in combination therapies on male vertebrae is not well understood. Since some differences in drug response have been observed between the genders [13-15, 23], the present work sought to determine whether administration of AMF alone or in combination with DXR would prevent any DOX-mediated bone damage and deterioration of vertebrae bone micro architecture in the young growing male rats.

## MATERIALS AND METHODS

### Drugs

Doxorubicin (DOX, Adriamycin™, Adria) and Dexrazoxane (DXR, Zinecard™, Pharmacia) were purchased from the SMBD-Jewish General Hospital pharmacy. Amifostine (AMF, Ethyol™) was a gift from MedImmune (The Netherlands).

### Animal Manipulation

All animal experiments were performed according to the guidelines of the Canadian Council on Animal Care. Lactating Sprague Dawley dams with 14 pups per female were purchased from Charles River Canada. At neonate day 10, rat pups were randomly divided into 7 groups of n=5 for saline, AMF+DOX, AMF+DOX+DXR, AMF, DOX, DXR and DOX+DXR treatments. Based on sexual maturity, 10 days of age in rats are more like boys prior to 6 years old. Pups were injected once intraperitoneally with either phosphate buffered saline (saline) or drugs: AMF (50 mg/kg), DOX (3 mg/kg), DXR (60 mg/kg), DXR+DOX (60 mg/kg + 3mg/kg), AMF+DOX (50 mg/kg + 3 mg/kg), or with AMF+DXR+DOX (50 mg/kg + 60 mg/kg + 3 mg/kg). AMF and DXR were injected 30 min prior to the DOX injection. The choice of the DOX and AMF concentrations is based on previous work in young rats [24] and accordingly to our previous work on female rats [22]. The concentration of DXR was 20-times the concentration of DOX, a ratio previously demonstrated to reduce DOX toxicity in adult rodent [25]. After injection, rat pups were returned to their mothers until weaning on neonate day 22. Rats were sacrificed on neonate day 38.

### Bone Mineral Density (BMD) and Bone Mineral Content (BMC) Analyses

Rats were sacrificed at day 38 (28 days post-injection). The PIXImus Bone Densitometer System #56069 (GE Lunar corporation) was used to measure bone densitometry using dual-energy X-Ray absorptiometry of bone tissue. The lumbar vertebraes were selected and their BMC (g) were measured by the PIXImus system. BMD (g/cm^2^) was obtained by the ratio BMC/bone area. Bone area, expressed in cm^2^, was the area selected to perform the BMC measurement.

### Micro Computed Tomography (microCT)

MicroCT data were acquired on a SkyScan T1072 X-Ray Microscope-Microtomograph (SkyScan, Aartselaar, Belgium). The vertebrae samples were placed vertically in a precision object manipulator located between the X-Ray source (a microfocus sealed X-Ray tube operating at a voltage of 100 kV and current of 98 μA) and the detector (an X-Ray charge-coupled device (CCD) camera). The samples were stepwise rotated around its axis, at a 0.9º angle. An image was acquired with exposition of 2,240 msec for every rotation step. A total of 206 images were obtained with a spatial resolution of 11.89 μm. The cross-sections along the specimen cylinder axis were reconstructed using Cone-Beam Reconstruction Software (SkyScan, Aartselaar, Belgium). Each cross-section was reduced in half size to facilitate the analysis, giving a voxel size of 21.89 µm. CTScan and 3D Creator software (both from SkyScan) were used to analyze and create 3-dimension rendering models respectively. The actual material volume was calculated using the segmentation method.

### Statistical Analyses

Analyses of variance (ANOVAs) and Fisher’s tests were performed using StatView software (SAS Institute, Cary, NC). Significant differences were retained at p<0.05.

## RESULTS

### Body Weight

The body weight of male rats was significantly reduced by treatment with DOX, DXR, AMF, DOX+AMF, and DOX+AMF+DXR (Table **1**). Treatment with DOX+DXR had no effect on body weight.

### Bone Mineral Content (BMC) and Bone Mineral Density (BMD) of Lumbar Vertebral Bodies

**Fig. (1):**
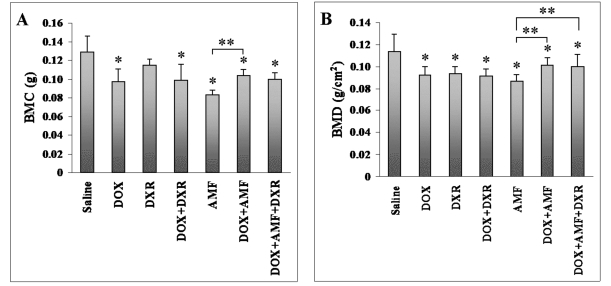
Effect of single-, double-, and triple combination treatments on the lumbar vertebrae BMC and BMD in male rats. BMC (**A**) and BMD (**B**) were measured in animals at sacrifice with a PIXImus Bone densitometer System. Data are expressed as the mean ± standard deviation (SD). *p<0.05 *vs* saline control; **p<0.05 *vs* AMF treatment.

BMC and BMD of the lumbar vertebrae were analyzed after treating male rats with different combinations of drugs (DOX, DXR, AMF, DOX+DXR, DOX+AMF, DOX+DXR+ AMF) and compared with the saline treatment (Fig. **1**). In general, drugs inhibited both BMC (Fig. **1A**) and BMD (Fig. **1B**). DOX treatment alone led to a significant 24% decrease in the BMC (p<0.01) as previously observed with female rats [22]. In order to better understand the protective effect of DXR, we decided to treat rats with DOX+DXR. Of special interest here was that DXR alone decreased BMC, but not significantly (p>0.05), by 11% (Fig. **1A**). DXR had no effect on the inhibition of BMC by DOX while the combination of DOX+DXR decreased the BMC by 24% (p<0.01), as observed with DOX alone. Treatment with AMF alone significantly decreased the BMC by 35% (p<0.01). Adding AMF to DOX did not lead to a significant improved BMC but led to a significant 18% decrease (p<0.01) compared to saline control. Furthermore, the triple combination DOX+DXR+ AMF did not lead to an improvement in the BMC, but to a decrease of 23% (p<0.01). Surprisingly, the BMC is 17% less (p<0.05) in AMF treated rats than in DOX+AMF treated rats, suggesting that the effect was not synergistic. Since the above drugs have effects on the body weight of the rats (Table **1**), we also normalized the results to the weight of the animals, but this did not change the results.

We next tested the effect of the same drug combinations on BMD. Interestingly, the BMD was significantly decreased by all drug combinations, including the DXR treatment alone (16%, p<0.01), when compared to the saline control (Fig. **1B**). DOX treatment decreased the BMD by 18% (p<0.01). When combined with AMF, the toxicity of DOX was reduced (11%, p<0.05), whereas when combined with DXR, there was no change in DOX toxicity that remained at 20% (p<0.01). The triple combination (DOX+DXR+AMF) was the least detrimental but BMD was still significantly decreased by 11% (p<0.05) when compared to the saline control. The most important BMD decrease was observed for AMF-alone treatment (24%, p<0.01), which was significantly lower than the DOX+AMF combination (11%, p<0.05) or the triple DOX+AMF+DXR combination (11%, p<0.05).

### Micro-Architecture of Lumbar Vertebral Bodies

To better understand the reduction in bone tissue, we also explore the effect of the different drug combinations on the vertebral bone architecture.

Fig. (**2**) shows the ventral view of the 3-dimension reconstruction of the male rat lumbar vertebrae obtained by MicroCT technique.

The trabecular bone (white rectangle) appeared slightly deteriorated by the following treatments: DXR, AMF, DOX+AMF, DOX+AMF+DXR. The quantitative analysis of the trabecular bone is presented in Table **2**. The transverse process (TP), which is the bony intrusion on either side of the arch of the vertebrae, was reduced by AMF and DOX+AMF treatments. Interestingly, the triple combination of DOX+DXR+AMF showed a transverse process very similar to that of the saline control. The different treatments seemed to have no or few effects on the accessory process (AP), which is a small apophysis at the base of the transverse process. However, this small structure is more difficult to analyze since a slight rotation of the vertebrae may have an important effect on the appearance of the structure.

**Fig. (3):**
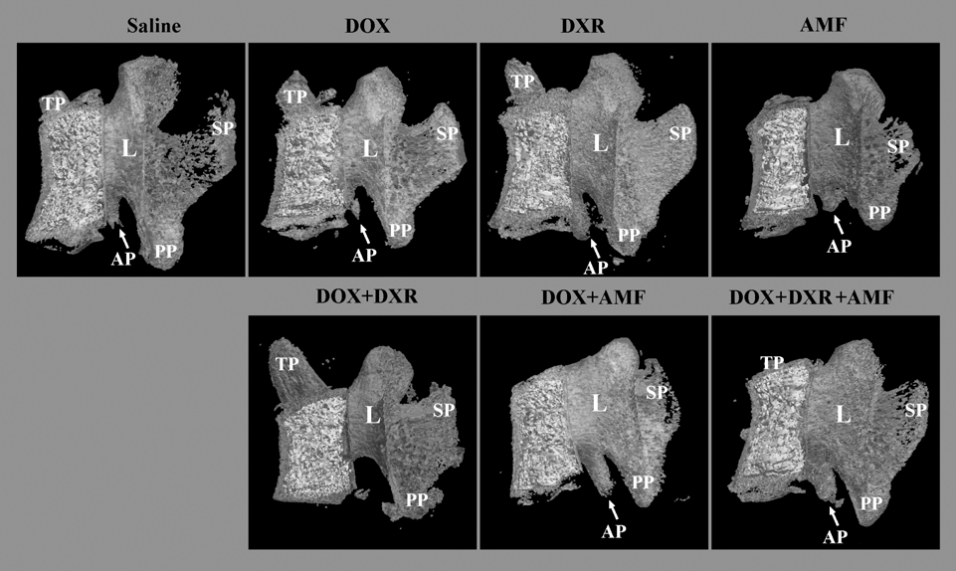
Effect of single-, double-, and triple combination treatments on the morphology of the lumbar vertebrae: a lateral view. The lumbar vertebrae were reconstructed in 3-dimension after micro computed tomography (micro CT) reconstruction and show the micro architecture of the lumbar vertebrae bone in the right lateral view. PP: posterior articular process; SP: spinous process; AP: accessory process; L: lamina.

A lateral view of the rat lumbar vertebrae was also created to highlight the effect of the different drug treatments on other parts of the rat vertebrae (Fig. **3**).

The right lateral view of the vertebrae showed that the injected drugs had no effect on the lamina (L) that forms the dorsal wall of the vertebral foremen, except with the DOX+AMF and DOX+DXR+AMF treatments where the concavity of this structure was not well defined. The effect of drugs on the spinous process (SP), which is the dorsal projection of the vertebral arch, was very similar to what was observed for the transverse process with a decrease by the AMF and DOX+AMF treatments while the triple combination of DOX+DXR+AMF had no effect. Again, the different treatments seemed to have no or few effects on the accessory process (AP) that is, as mentioned before, more difficult to analyze. Finally, the different treatments had no effect on the posterior articular process (PP) of the male rat vertebrae.

Table **1** summarizes the quantitative results generated from MicroCT of trabecular bone of the lumbar vertebral bodies of male rats. The relative bone volume/tissue volume ratio (BV/TV) of the vertebral bodies was not affected by DOX, AMF, DOX+AMF, and DOX+DXR+AMF treatments. However, there was a non-significant trend toward decreased BV/TV with DXR and DOX+DXR (24 and 19%, respectively), when compared to the saline control. In contrast, the BV/TV increased when rats were treated with DOX+AMF (1.53 fold, p<0.05) when compared to DXR treatment and tended to increase (1.44 times, p=0.07) when compared to DOX+DXR treatment. The bone surface/bone volume (BS/BV) ratios were not affected by the different treatments. The trabecular number (Tb.N) was decreased with DXR (19%, p<0.05) and tended to decrease with DOX+DXR (15%, p<0.09), when compared to saline control. Tb.N was also lower in DXR-treated rats compared to DOX-treated rats (22%, p<0.05). DOX+AMF increased the Tb.N of male rats when compared to the DXR treatment (1.37 times, p<0.05) and tended to increase this Tb.N when compared to the DOX+DXR-treated animals (1.30 times, p=0.06).

The trabecular separation (Tb.Sp), which is the thickness of the marrow spaces between trabecular structures, was not significantly affected by the different treatments, except for the DOX+AMF treatment that significantly decreased Tb.Sp by 11% (p<0.02) and 14% (p<0.005) when compared to DXR and DOX+DXR treatments, respectively.

## DISCUSSION

Children with cancers may be more highly susceptible to secondary toxicities than adults because they are actively growing [20]. The increased numbers of childhood cancer survivors is a consequence of improvement in cancer therapies which allow more children to survive their cancer. These same therapies can reduce bone mineralization, reduce final height and sitting height of cancer survivors and increase the incidence of fracture [7, 8]. At high cumulative dose (550 mg/m^2^), DOX increases dramatically the risks of developing cardiac side effects, including congestive heart failure, dilated cardiomyopathy, and death [13-15]. Reactive oxygen species, generated by the interaction of doxorubicin with iron, can also damage the myocytes, causing myofibrillar loss and cytoplasmic vacuolization [11, 12]. However, little is known about the indirect or direct effects of anti-cancer drugs, such as DOX, and of cytoprotective agents, such as DXR and AMF, on the spine. Moreover, the effect of DOX, with or without DXR and/or AMF, on bone of children is not well understood. We showed recently that a single injection of DOX in young female rats is associated with low bone turnover resulting in vertebrae bone growth deficits [22, 23]. However, little work has been done to investigate the effect of these drugs on bone mass of the male vertebrae even though some differences in drug response between the genders have been observed [13-15, 23]. Some studies suggest that young females have a greater incidence for DOX-induced cardiotoxicity than young males [13-15] although a gender bias was not detected in other studies [10, 25]. The aim of this study was to determine whether administration of AMF and/or of DXR would prevent any DOX-mediated bone damage in the young growing male rats.

The present data indicate that DOX selectively decreased BMD and BMC accrual in the lumbar vertebrae of male rats and that neither DXR nor AMF were able to prevent these reductions. Furthermore, AMF treatment alone appears to be worse than DOX treatment. This is contrary to what was expected and to what was found in a previous study using knees of rats. In this study, seventy-two weanling rats had their right knee irradiated with single fraction 17.5 Gy, whereas the left leg was used as an internal control [26]. The results indicated that the animals receiving AMF had BMD that was closer to controls only adjacent to the chondroosseous junction at 0.5, 2, and 3 weeks. Similarly, AMF had a significant protective effect against DOX-induced early alopecia in young rats [27]. However, in this study AMF did not protect young rats against the late toxic effects of DOX on linear growth, body weight, plasma leptin levels, and heart or testicular tissue. AMF treatment may therefore not always protect from DOX toxicity. Moreover, the decrease of both BMC and BMD by AMF is not sex-dependent since we observed the same tendency in our previous studies in females [22], whereas in this case, the BMD and BMC values were most significantly decreased by DOX treatment. Further studies are necessary to determine if these changes correlate with changes in serum markers of bone turnover such as alkaline phosphatase and C-telopeptides. These results have implications in relation to the safety of AMF treatment and its mechanisms of action before wider clinical use of this drug in pediatric cancer patients is recommended. As AMF is not clinically used on its own, further studies are needed to better characterize the effects of AMF on bone growth, mineral density, and architecture. Moreover, long-term evaluation will be necessary to determine the possible reversibility of the effect of these drugs on bone architecture.

Micro-architecture data in male rats treated with DXR and with the two double combinations treatments are very interesting. It has been shown that decreases in the BV/TV ratio and in Tb.N accompanied with an increase in Tb.Sp were correlated with bone fracture severity in vertebrae of osteoporotic women [3]. In the present study, DXR treatment negatively affected the trabecular bone structure, with a significant effect on Tb.N and a mild effect on BV/TV. This suggests an increased risk of fracture in the DXR-treated animals. This remains to be investigated with biomechanical studies. This effect may not be due to the DNA topoisomerase II activity. Both DOX and DXR reduce DNA topoisomerase II activity.

PIXImus analyzes simultaneously the properties of both the cortical and the trabecular bones, without discrimination (Fig. **1**). Micro CT analyzes only the characteristics of the trabecular bone (Table **2**). Since, in the present study, both cortical and trabecular bone characteristics measured by the PIXImus (BMD and BMC) were significantly affected by drug treatments, whereas the trabecular bone characteristics alone (measured by micro CT) was less significantly affected by the same drugs, it appears that in male rats, the cortical bone was the most affected by the single-, double-, and triple-therapies. On the contrary, the age-matched female rats we previously studied [22] showed significant defects for all drugs tested in both lumbar vertebrae BMD/BMC and micro-architecture. This might be explained by sex effects. In humans, BMD values in lumbar spines of girls have been shown to be higher when compared with age-matched boys. Boys showed higher BMD values preferentially for cortical sites [13], probably affecting as well the micro-architecture at these sites. However, since girls and boys mature at different rates, further studies are necessary to definitively conclude on the sex-dependent effect of these drugs in human.

The mechanisms for sex specificity are not clear. We speculate that the sex-dependent specificity of DOX, DXR and AMF on bone tissue could be, at least in part, caused by sex steroid hormone levels. Although this hypothesis remains highly speculative and requires further investigations, differences in sex steroids remains a potential mechanism for differences in the response of these pre-pubertal males and females. It must be noted that, the level of sex steroid hormones is likely very low in day 10 neonate rats, the time of injection in this study. Nevertheless, this information would be useful in designing better cancer combination therapies that do not lead to bone deterioration.

## Figures and Tables

**Fig. (2):**
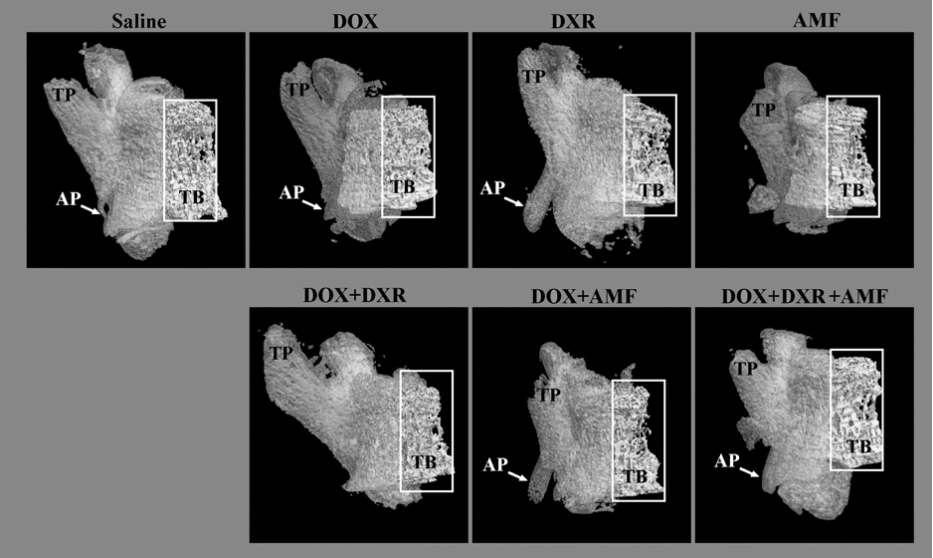
Effect of single-, double-, and triple combination treatments on the morphology of the lumbar vertebrae: a ventral view. The lumbar vertebrae were reconstructed in 3-dimension after micro computed tomography (micro CT) reconstruction and show the micro architecture of the lumbar vertebrae bone in the ventral view. The trabecular bone (TB) is presented in the white boxes. TP: transverse process; AP: accessory process.

**Table 1:** 

Drug	Body Weight at Sacrifice (g)	Significant Difference *vs* Saline
Saline	171 ± 7	-
DOX	133 ± 9	p < 0.001
DXR	145 ± 10	p < 0.001
AMF	139 ± 7	p < 0.001
DOX+DXR	173 ± 8	p > 0.05
DOX+AMF	148 ± 8	p < 0.001
DOX+DXR+AMF	152 ± 10	p = 0.001

**Table 2:** 

	BV/TV (%)	BS/BV (mm^-1^)	Tb.N (mm^-1^)	Tb.Sp (mm)
Saline	21.5 ± 3.4	57.9 ± 7.5	2.3 ± 0.3	0.25 ± 0.01
DOX	22.3 ± 1.5	56.6 ± 1.0	2.4 ±0.1	0.25 ± 0.01
DXR	16.3 ± 1.9	65.5 ± 4.6	1.8 ± 0.2§	0.26 ± 0.01
DOX+DXR	17.3 ± 2.9	62.9 ± 5.0	1.9 ± 0.2	0.27 ± 0.01
AMF	21.9 ± 5.1	57.5 ± 7.8	2.3 ± 0.4	0.25 ± 0.02
DOX+AMF	24.9 ± 5.8*	53.6 ± 9.3	2.5 ± 0.4*	0.23 ± 0.01*‡
DOX+AMF+DXR	21.5 ± 4.1	58.0 ± 6.4	2.3 ± 0.4	0.25 ± 0.01
